# Macrophage Paracrine Signalling Differentially Affects Fibroblast-Induced Collagenous Tissue Remodelling

**DOI:** 10.1007/s13770-025-00766-1

**Published:** 2025-11-28

**Authors:** Hannah F. M. Brouwer, Amal K. Mansoor, Sylvia Dekker, Carlijn V. C. Bouten, Keita Ito, Jasper Foolen, Anthal I. P. M. Smits

**Affiliations:** 1 Department of Biomedical Engineering, Eindhoven University of Technology, PO Box 513, 5600 MB Eindhoven, The Netherlands; 2 Institute for Complex Molecular Systems (ICMS), Eindhoven University of Technology, 5600 MB Eindhoven, The Netherlands

**Keywords:** Macrophage polarisation, Tissue engineering, Collagen remodelling

## Abstract

**Background:**

Upon injury, tissue repair often leads to a loss in functionality, organisation, and structure. The immune system, particularly macrophages, is crucial during tissue healing. Macrophages polarise into pro-inflammatory M1 and anti-inflammatory M2 subsets, regulating various stages of tissue healing. Macrophages steer fibroblasts in the process of extracellular matrix degradation, synthesis, and rearrangement. However, the direct role of paracrine signalling by different macrophage phenotypes on fibroblast-induced structural tissue remodelling remains elusive. Therefore, this study aimed to explore how paracrine factors from M1, M2a, and M2c macrophages affect fibroblast remodelling abilities in an* in vitro* model system.

**Methods:**

Macrophages were polarised* in vitro*, and their conditioned medium or cytokine-enriched medium containing specific macrophage-secreted factors was added to fibroblast-populated reconstituted collagen tissues.

**Results:**

Macrophage-conditioned media led to changes in fibroblast-induced tissue compaction for all macrophage subsets. The presence of macrophage polarising factors in the conditioned medium, particularly LPS/IFNγ, and high serum levels directly affected tissue compaction and matrix remodelling gene expression. Without these confounding factors, M1 cytokine-enriched medium led to reduced tissue compaction when compared to M2a/M2c cytokine-enriched media. MMP activity analysis showed that matrix degradation likely contributed to tissue compaction.

**Conclusion:**

Factors secreted by M1 macrophages resulted in reduced tissue compaction compared to M2a/M2c macrophages in an* in vitro* model of tissue remodelling, suggesting a diminished capacity for fibroblasts to remodel the extracellular matrix. Importantly, factors to polarize macrophages and serum are regarded as confounding factors in studying the effect of paracrine signalling by macrophages on tissue remodelling.

**Supplementary Information:**

The online version contains supplementary material available at 10.1007/s13770-025-00766-1.

## Introduction

After tissue injury, the repair process is complex and multifaceted, aiming to restore both the architecture and function of the affected tissue [1]. One common limitation of tissue repair is the inability to fully regain original strength, organisation, and functionality. This loss of tissue structural integrity is often seen as a failure of functional tissue remodelling due to an imbalance between extracellular matrix (ECM) degeneration and synthesis. In tendon healing, for example, tendons often heal by depositing excessive, disorganised ECM, which is in contrast to the naturally present strong tissue anisotropy [2]. Similarly, in skin, improper tissue repair creates hypertrophic scars or keloids, which are painful, create movement restrictions, and are often aesthetically undesirable [3, 4]. This failure in proper matrix remodelling can result in mechanically inferior tissue, pain, decreased function, and eventual risk of (re-)rupture. The remodelling process can evolve into a progressive, irreversible, and excessive accumulation of tissue matrix, leading to fibrosis and permanent scarring [5]. Understanding the mechanisms behind the inability to restore tissue integrity is therefore pivotal. One of the key cellular players in matrix remodelling is the fibroblast, which is responsible for degrading, depositing, and organising the ECM, specifically collagen. Fibroblast behaviour is known to be influenced by various microenvironmental cues, but also by neighbouring cells, such as macrophages [6].

Tissue repair, following an injury, progresses through three distinct phases: inflammation, proliferation, and remodelling. Macrophages, as key regulatory immune cells, play an integral role throughout these phases. Depending on local environmental cues, such as cytokines and/or danger signals, macrophages can reversibly polarise over a spectrum of different phenotypes, with the pro-inflammatory “M1” phenotype and the pro-wound healing “M2” phenotype at the phenotypical extremes [7, 8]. M1 macrophages are typically considered to arise from the presence of pro-inflammatory cytokines, such as interferon-γ (IFN-γ), as well as damage- or pathogen-associated molecular patterns. Their primary function is to attack and clear up threats by secreting pro-inflammatory signals and destructive moieties, such as reactive oxygen species [9, 10]. M2 macrophages, on the other hand, are a diverse set of macrophages that are generally considered to be important for stimulating matrix repair and regulating inflammation. Although no standardized nomenclature exists and heterogeneities within populations are present, an important functional distinction is between interleukin-4 (IL-4)-induced ‘M2a’ macrophages and IL-10-induced ‘M2c’ macrophages, which primarily promote tissue production and regulate inflammation and tissue remodelling, respectively [11–13]. It is known that macrophages influence fibroblast behaviour through cytokine secretion. Pro-inflammatory cytokines, such as IL-1 stimulate the proliferation of fibroblasts, and TNF-α can inhibit collagen synthesis and stimulate collagenase synthesis by fibroblasts [14–17]. On the other hand, anti-inflammatory factors such as TGF-β drive ECM synthesis by upregulating ECM-related genes and downregulating genes associated with ECM degradation [18]. Hence, these signalling factors can steer fibroblast behaviour towards ECM degradation or deposition. Furthermore, the ability of the fibroblasts to rearrange their surrounding matrix and subsequently align the collagen fibres by exerting tractional forces is affected by factors such as TGF-β [19]. If fibroblasts lose their ability to exert tractional forces and reorganize collagen fibres, the structural integrity of the resulting repaired tissue is impaired [20, 21]. By coordinating fibroblast activity and ECM remodelling, macrophages thus assist in maintaining the integrity of the repaired tissue.

Despite the relevance of macrophages in guiding fibroblast phenotype and functioning during tissue repair, remarkably little is known about how the different types of macrophages can influence the ability of fibroblasts to organise collagen fibres. Specifically, if and how paracrine factors secreted by inflammatory M1 macrophages, and pro-fibrotic M2a and inflammation-suppressing M2c macrophages, affect the structural remodelling abilities of fibroblasts remains elusive [22, 23]. To explore these effects, reconstituted collagen tissues, embedded with fibroblasts, were utilised as an *in vitro* model. These constructs are particularly well-suited for replicating aspects of tissue repair at the cellular and ECM level, offering the possibility to study how fibroblasts reorganise their structural environment [24, 25]. Since *in vivo* remodelling involves degradation, production, and (re-)arrangement of the ECM, the ability of cells to compact collagen-rich constructs *in vitro* can serve as a model for their tissue remodelling potential [26]. Macrophages were chemically polarised *in vitro* towards defined subsets, and the conditioned medium obtained from the macrophages was then added to the fibroblast-collagen constructs. The effect of conditioned media from differently polarised macrophages and the effect of specific soluble factors, as detected in the conditioned media of the different macrophage subsets, on tissue remodelling was tested. It was hypothesised that paracrine factors from M1 macrophages impair fibroblast-mediated ECM compaction by increasing ECM degradation and disrupting their contractile abilities, whereas M2a and M2c paracrine factors would promote these remodelling abilities. Ultimately, we aimed to unravel the effects of differently polarised macrophages on tissue compaction as a measure of tissue remodelling, to enable adequate guidance of the tissue healing process towards functional healing.

## Materials and methods

### Production of macrophage conditioned medium

#### THP-1 cell culture and macrophage polarization

**Fig. 1:**
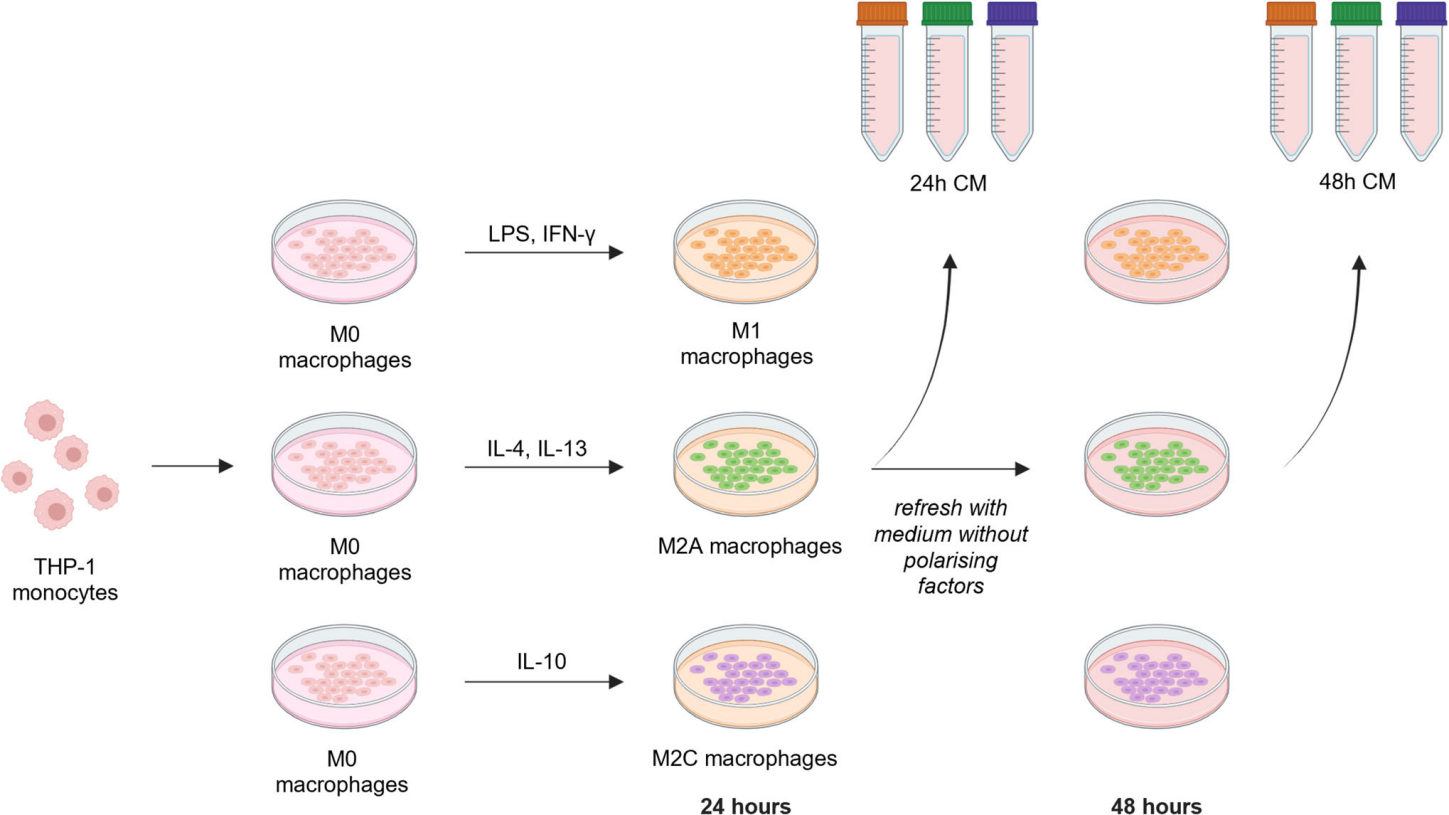
Graphical representation of THP-1 monocyte culture, polarised towards M1, M2a and M2c macrophages. Macrophage conditioned medium was collected 24 and 48 h after starting polarization and stored at -80 °C. Created with BioRender.com

Cells from a human monocytic cell line (THP-1; LGC Group, ATCC-TIB-202, Teddington, England) were cultured in expansion medium, i.e., Roswell Park Memorial Institute (RPMI) 1640 Medium (11875093, Thermo Fisher Scientific, Waltham, MA, USA) with 10% fetal bovine serum (FBS; S-FBS-SA-015, Serana, Brandenburg, Germany) and 1% penicillin/streptomycin (15140148, Thermo Fisher Scientific) in suspension, at 37 °C and 5% CO_2_. Medium was refreshed three times a week to keep cell densities consistent (0.5–1.5 × 10^6^ cells per mL of medium). Cells at P24 and P26 were seeded at a density of 1.5 × 10^5^ per cm^2^ and stimulated with phorbol 12-myristate-13-acetate (PMA; P8139, Sigma-Aldrich, St. Louis, MO, USA) for 2 days to become adherent macrophages. Medium was then switched back to expansion medium for 24 h, and subsequently the macrophages were stimulated with different polarising factors (PF) for 24 h (Fig. 1). Polarising factors commonly reported in literature were used to polarise the macrophages [27]. For M1 polarization, 100 ng/mL lipopolysaccharides from *Escherichia coli O111:B4* (LPS; Sigma-Aldrich, L5293) and 20 ng/mL IFN-γ (300–02, PeproTech, Thermo Fisher Scientific) were added to expansion medium. For M2a polarization, 20 ng/mL IL-4 (200–04, PeproTech) and 20 ng/mL IL-13 (200–13, PeproTech) were added. For M2c polarization, 50 ng/mL IL-10 (200–10, PeproTech) was added.

#### Collection and analysis of macrophage-conditioned medium

After 24 h of polarization, the medium was collected. Due to the possible presence of PFs still after 24 h that could influence our read-outs, fresh expansion medium without PFs was added to the cells, and after another 24 h in expansion medium (48 h in total from start polarization), the medium was again collected (Fig. 1). Consequently, the medium collected at 24 h contained both the added PFs and cell-secreted factors, while the 48-h medium only contains factors that are secreted between 24 and 48 h. Collected medium was centrifuged (150 g, 5 min) and stored at -80 °C until use. This procedure was repeated three times (n = 3).

To analyse the contents of the conditioned media, Multiplex ELISA was performed at the Multiplex core facility of the Laboratory for Translational Immunology of the University Medical Center Utrecht, The Netherlands, as described by de Jager et al. [28]. Specific proteins were selected that are characteristic of macrophage phenotype, including the PFs, and that have an expected role in macrophage-fibroblast signalling (Table 1). Specifically, we measured the pro-inflammatory cytokines Tumor Necrosis Factor-α (TNF-α) and IL-12, which are characteristic for M1 macrophages, Monocyte Chemoattractant Protein-1 (MCP-1) and IL-8, which are important cytokines for the recruitment of monocytes and neutrophils, respectively, IL-1β and IL-18 representing the products of the inflammasome, TGF-β as one of the main fibrogenic growth factors secreted by macrophages, and the polarizing factors IFN-γ, IL-4 and IL-13, and IL-10, for M1, M2a and M2c macrophages, respectively [27, 29, 30]. The measured protein concentrations were plotted as the mean of the three repetitions. In case an out-of-range (OOR) value was measured, the upper or lower cut-off value of that specific protein was taken (Supplementary Tables 1 and 2).

**Table 1:** Proteins analysed via multiplex enzyme-linked immunosorbent assay

Protein	Symbol	Function	Refs
Interferon gamma	IFN-γ	Th1/M1-associated cytokine, promotes M1 macrophage polarization; inhibitor of collagen production and fibroblast proliferation	[27, 43, 44]
Interleukin 4	IL-4	Th2/M2-associated cytokine, promotes M2a macrophage polarization; promotes myofibroblasts and collagen production	[27, 44, 47, 48]
Interleukin 13	IL-13	Th2/M2-associated cytokine, promotes M2a macrophage polarization; promotes myofibroblasts and collagen production	[27, 44, 47]
Interleukin 10	IL-10	Treg/M2-associated cytokine, promotes M2c macrophage polarization; inhibitor of collagen production	[27, 45, 46]
Tumour necrosis factor alpha	TNF-α	M1-associated cytokine; regulator of apoptotic cell death; stimulus for collagen production	[29, 55]
Interleukin 12 p70	IL-12 p70	M1-associated cytokine; induces IFN-γ production	[60]
Interleukin 1 beta	IL-1β	M1-associated cytokine, product of NLRP3 inflammasome; regulates cell proliferation, differentiation and apoptotic cell death; stimulates fibrosis via TGF-β	[29, 30, 55]
Interleukin 18	IL-18	M1-associated cytokine, product of NLRP3 inflammasome; implicated with fibrosis	[30]
Interleukin 8	IL-8	Chemoattractant for neutrophils; stimulates migration of fibroblasts and loss of focal adhesions	[57, 58]
Monocyte chemoattractant protein 1	MCP-1	Chemoattractant for monocytes/macrophages; stimulates TGF-β production by fibroblasts	[56, 59]
Transforming growth factor beta 1	TGF-β1	Key stimulus for myofibroblast activation and collagen synthesis	[52, 53]

#### Producing cytokine-enriched media

Based on the results of the multiplex-ELISA, defined cytokine-enriched media (‘CE’) were produced to mimic the conditioned media for the different macrophage subsets. For this, the measured concentrations of the secreted factors in the 24 h medium were used (Table 4) to manually create the different mixtures of medium. This was done for culture medium containing 10% FBS (Serana) and 2.5% FBS (Serana). In addition, to understand the direct influence of the polarizing factors on fibroblast behaviour, culture medium with only polarizing factors was created (‘PF’). These media were prepared and placed in the incubator for 24 h before being added to the tissues to account for the half-life of the factors. Lastly, polarizing factors were added to the cytokine-enriched media (‘CE + PF’) in order to compare these to the 24 h medium. An overview of all media used in this study can be found in Table 2.

**Table 2:** Overview of media used in this study. RPMI expansion medium was always used as the control

Control		RPMI expansion medium
24 h CM	M1	collected 24 h after stimulation with LPS and IFN-γ
	M2a	collected 24 h after stimulation with IL-4 and IL-13
	M2c	collected 24 h after stimulation with IL-10
48 h CM	M1	collected 48 h after stimulation with LPS and IFN-γ and refreshing with expansion medium
	M2a	collected 48 h after stimulation with IL-4 and IL-13 and refreshing with expansion medium
	M2c	collected 48 h after stimulation with IL-10 and refreshing with expansion medium
CE medium	M1	Expansion medium with TNF-α, IL-1β, IL-12, IL-8, IL-18, MCP-1, TGF-β
	M2a	Expansion medium with TNF-α, IL-1β, IL-12, IL-8, IL-18, MCP-1, TGF-β
	M2c	Expansion medium with TNF-α, IL-1β, IL-12, IL-8, IL-18, MCP-1, TGF-β
PF medium	M1	Expansion medium with LPS and IFN-γ
	M2a	Expansion medium with IL-4 and IL-13
	M2c	Expansion medium with IL-10
CE + PF medium	M1	Expansion medium with M1 CE and PFs combined
	M2a	Expansion medium with M2a CE and PFs combined
	M2c	Expansion medium with M2c CE and PFs combined

24 h Conditioned Medium (CM) and 48 h CM were obtained directly from macrophages as shown in Fig. 1. Cytokine-enriched media were made based on the multiplex ELISA measurements as shown in Table 4, containing either macrophage secreted factors only (CE), polarizing factors only (PF), or the secreted factors in combination with macrophage polarizing factors (CE + PF)

### Creating collagenous tissues

#### Model system

**Fig. 2:**
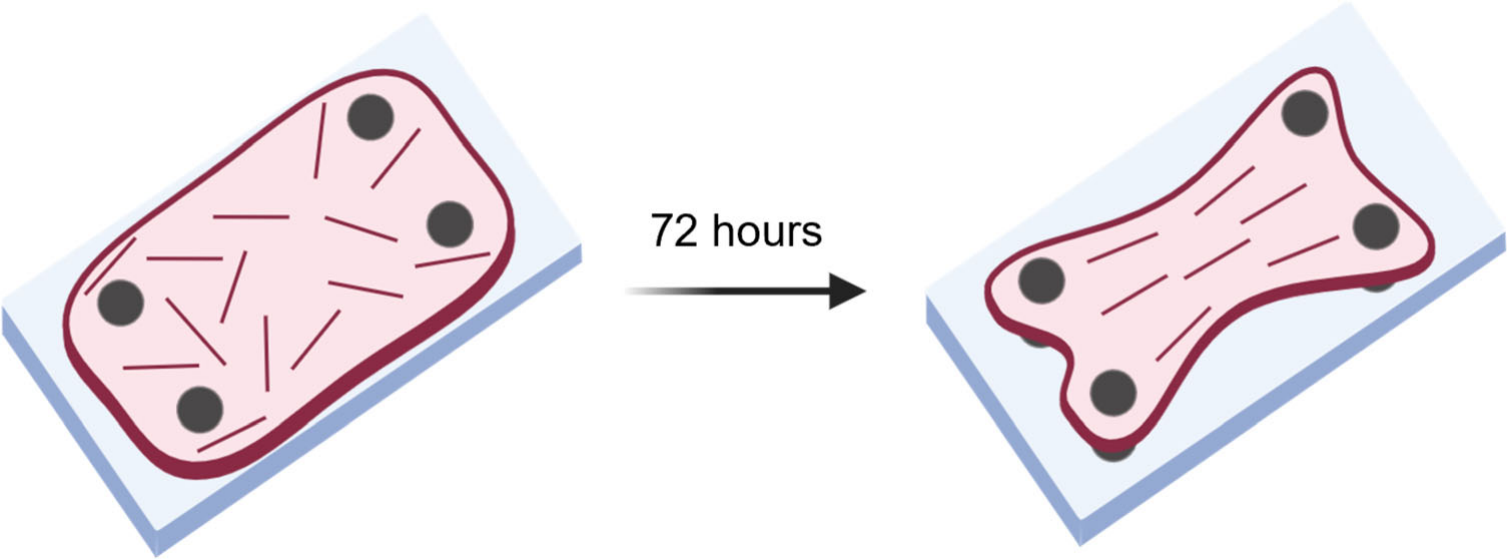
Graphical representation of the tissue model system. Collagen gel (pink) containing randomly oriented fibroblasts (red). Over a period of 72 h the cells compact the collagen around 4 posts (grey) and align along the longitudinal axis. Created with BioRender.com

A previously designed model system was used to create cell-populated reconstituted collagen tissues [25]. Briefly, cell-populated reconstituted collagen matrices were produced via anchorage around 2 × 2 silicone posts, resulting in uniaxially constrained tissues that aligned over time due to the cells compacting the tissues (Fig. 2). Silicone rubber (Dragon Skin; Smooth-On, Easton, PA, USA) posts were mounted on a polydimethylsiloxane (PDMS, a Sylgard 184 Silicone Elastomer Kit, a prepolymer and curing agent, a 10:1 weight ratio, Dow Corning, Midland, MI, USA) substrate and stored at room temperature until use. Before use, the post systems were sterilised by covering the well plates in 70% EtOH and allowing evaporation overnight. Subsequently, the systems were coated with Pluronic F-127 (P2443, Sigma-Aldrich) to render the surface non-adherent, and thus to prevent the tissues from attaching.

#### Fibroblast culture

Normal human dermal fibroblasts (NHDF; CC-2511, Lonza, Basel, Switzerland) were used as a contractile cell. Human dermal fibroblasts were used as the cell source due to their wide availability and easy harvestability. NHDFs were thawed and expanded to P6 at 3333 cells per cm^2^ in Dulbecco’s Modified Eagle’s Medium (DMEM; Gibco, 41966, Thermo Fisher Scientific) with 10% FBS (Serana) and 1% penicillin/streptomycin (Thermo Fisher Scientific) at 37 °C and 5% CO_2_. Medium was refreshed after three days. After reaching sub-confluency after 4 days, NHDFs were detached using 0.05% trypsin/Ethylenediaminetetraacetic acid (EDTA) with phenol red (25300–054, Gibco) and embedded in collagen gels.

#### Preparing and culturing reconstituted collagen tissues

A gel mixture of culture medium, rat tail collagen type I (5056, Advanced Biomatrix, Carlsbad, CA, USA) and 1 M NaOH was made with a final collagen concentration of 1 mg/mL. NHDFs were mixed with the gel at 1 million cells per mL gel. The cell-gel mixture was subsequently added to the model system and left to polymerise for 45 min in an incubator at 37 °C, after which the different types of conditioned medium were added to each well (4 mL). After 72 h of culturing, tissues were released from the posts and directly put in liquid nitrogen and stored at -80 °C for RNA isolation. Alternatively, tissues were fixed in 3.7% formaldehyde solution in PBS for 30 min, while anchored to the posts for microscopy purposes. Medium was collected, centrifuged (250 g, 5 min) and the supernatant was stored at -80 °C for further analysis.

### Tissue analyses

#### Tissue compaction

**Fig. 3:**
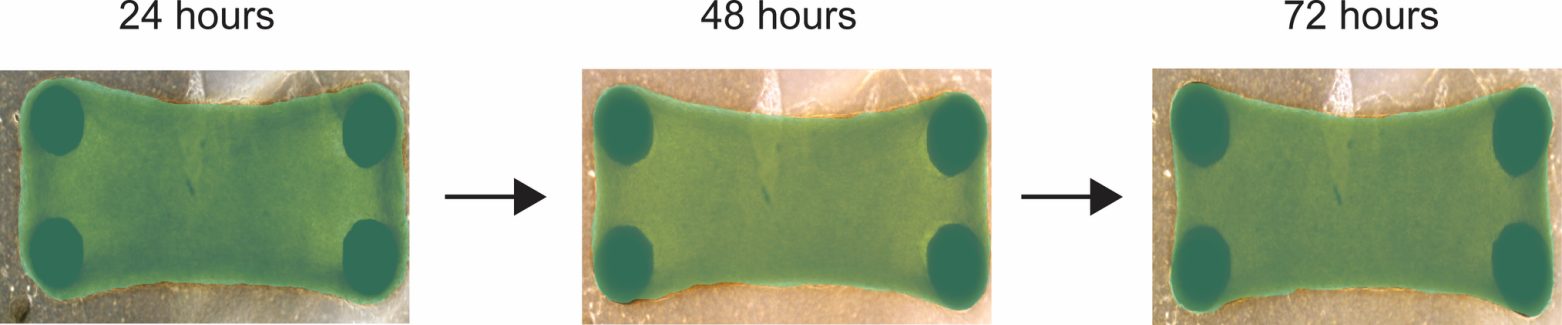
Brightfield images of a single tissue followed over time (24, 48, 72 h). The surface area masked in green was used to calculate the percentage decrease in surface area as a measure of compaction

Brightfield images were made after 24, 48 and 72 h using an EVOS™ XL Core microscope, capturing each tissue completely in a single image. Masks of the tissues in the images were drawn manually using Cellpose [31], after which the surface area of each tissue was calculated using an in-house developed ImageJ [32] script. From the surface areas at the three different timepoints of each tissue, the percentage decrease in surface area was used as a measure of the compaction of the tissue (Fig. 3).

#### Fibroblast gene expression

Tissues were taken from the -80 °C freezer and lysed by adding 600 µL RLT-buffer with 2-mercaptoethanol (M6250, Sigma-Aldrich). For each measurement, 3 technical replicates were pooled to obtain sufficient RNA, and a total of 3 independent experiments were done and used for analysis. RNA was isolated using the Qiagen RNeasy kit (Qiagen, Venlo, Netherlands) according to the manufacturer’s protocol. Purity and quantity of the RNA was determined with a spectrophotometer (Nanodrop, ND1000, Isogen Life science, IJsselstein, Utrecht, Netherlands). All the samples were diluted to the same RNA concentration of 9 ng/µl. Subsequently, cDNA was synthesised in 20 µl reaction volume, containing dNTPs (Invitrogen, Thermo Fisher Scientific), random primers (Promega, Madison, WI, USA), Superscript 4 reverse transcriptase (Invitrogen), Dithiothreitol (DTT, Invitrogen) and RNA-free water. A Thermal Cycler (C1000 Touch, Bio-rad, Hercules, CA, USA) was used for the cDNA synthesis with the following heating steps: 65 °C for 5 min, 23 °C for 10 min (during which the SSIV was added), 50 °C for 30 min, 80 °C for 10 min, and finally 12 °C for cooling down. The samples were then checked for contaminations by performing PCR with a primer of the housekeeping gene Glyceraldehyde-3-phosphate dehydrogenase (GAPDH). The cDNA samples were stored at -20 °C until qPCR was performed. Primers of genes involved in tissue production, degradation and contraction (Table 3) were, together with SYBR Green Supermix (Bio-Rad) and ddH2O, used for real-time qPCR (CFX384 Touch Real-Time PCR Detection System; Bio-Rad). GAPDH, ATP, B2M, β-ACT, HPRT and TOP1 were first tested as reference genes using reffinder [33]. β-ACT was determined to be the most stable for all the test conditions in the experiments where conditioned medium was refreshed after 24 h and GAPDH was determined to be the most stable in the other experiments (Supplementary Fig. 1). All samples were diluted 100 times and analysed in duplicates. The following thermal protocol was used: 95 °C for 3 min, a cycle of each 95 °C for 20 s, 60 °C for 20 s and 72 °C for 30 s repeated 40 times, 95 °C for 1 min, 65 °C for 1 min, and finally a melting curve measurement. The DDCt- or Livak method was used to analyse the data. Gene expression for the samples was normalized to the reference gene and, afterward, to the mean of the control RPMI group.

**Table 3:** Quantitative polymerase chain reaction primers for production, degradation and contraction

Readout	Primer	Symbol	Amplicon size (bp)	Accession number	Primer sequence [5′-3′]
Production	Collagen 1	Col I	112	NM_000088	FW: AATCACCTGCGTACAGAACGG RW: TCGTCACAGATCACGTCATCG
	Collagen 3	Col III	140	NM_000090	FW: ATCTTGGTCAGTCCTATGC RW: TGGAATTTCTGGGTTGGG
	Decorin	Dcn	91	NM_133503	FW: AATGCCATCTTCGAGTGGTC RW: TGCAGGTCTAGCAGAGTTGTGT
Degradation	Matrix metallo-proteinase 1	MMP-1	101	NM_001145938.1	FW: CGCACAAATCCCTTCTACCC RW: CTGTCGGCAAATTCGTAAGC
	Matrix metallo-proteinase 2	MMP-2	174	NM_001127891.2	FW: ATGACAGCTGCACCACTGAG RW: ATTTGTTGCCCAGGAAAGTG
	Tissue inhibitor of metallo-proteinase 1	TIMP-1	102	NM_003254.2	FW: TGACATCCGGTTCGTCTACA RW: TGCAGTTTTCCAGCAATGAG
	Tissue inhibitor of metallo-proteinase 2	TIMP-2	104	NM_003255.4	FW: GGAGGAATCGGTGAGGTC RW: AACAGGCAAGAAGCAATGG
Contraction	Alpha smooth muscle actin	α-SMA	134	NM_001613.1	FW: CGTGTTGCCCCTGAAGAGCAT RW: CCGCCTGGATAGCCACATACA

#### Immunofluorescent microscopy

Whole-mount staining was performed on fixed tissues. Tissues were gently removed from the posts and permeabilized for 30 min with 0.5% Triton X-100 (Merck, Schipholrijk, The Netherlands) and washed three times for 5 min with PBS. For each condition, one sample of each repetition was stained with Phalloidin-Atto (Sigma-Aldrich, 65906) for 1 h to visualise F-actin, and with 4′,6-diamidino-2-phenylindole dihydrochloride (D9542, Sigma-Aldrich) for 10 min to visualize the nuclei. Prior to staining with phosphorylated myosin light chain (3675S, Cell Signaling, Danvers, MA, USA) and alpha smooth muscle actin (a2547, Sigma-Aldrich), tissues were blocked with 4% goat serum in 0.05% Tween in PBS for one hour at room temperature. After washing thrice with 0.4% goat serum in 0.05%Tween in PBS, the Phospho-Myosin Light Chain 2 antibody, diluted in 0.4% goat serum in 0.05% Tween in PBS at 1:200, was added and incubated overnight at 4 °C. After washing thrice, the secondary antibody, anti-mouse IgG1 Alexa 488 (A21121, Molecular Probes, Eugene, OR, USA), diluted at 1:200 in PSB, was added and incubated for an hour at room temperature. Finally, all the tissues were washed three times for 5 min with PBS before imaging. Z-stacks were taken in the middle of each tissue with a confocal microscope (Leica TCS SP5X; Leica Microsystems CMS GmbH, Mannheim, Germany) to visualize the cell distribution and cell morphology within the tissues.

#### Collagen zymography

The presence of collagenases in the tissue culture medium collected after 72 h was detected by collagen zymography. Zymography was performed by loading the same volume of medium (15 µL plus 5 µL 4 × Laemmli sample buffer) for each group and culture medium with the same serum amount as the control. An 8% SDS polyacrylamide gel (30% Acrylamide/bis solution 37.5:1, Bio-Rad) was created containing rat tail collagen type I (5056, Advanced Biomatrix, final concentration 0.67 mg/ml). Following electrophoresis, gels were soaked twice for 30 min with washing buffer (50 mM TRIS/HCl pH 7.5, 2.5% Triton X-100, 5 mM CaCl2, 1 µM ZnCl2) to remove the SDS. Gels were incubated overnight at 37 °C in substrate buffer (50 mM TRIS/HCl pH 7.5, 1.0% Triton X-100, 5 mM CaCl2, 1 µM ZnCl2) to activate the MMPs. After incubation, gels were stained overnight in a Coomassie Blue (Sigma-Aldrich) staining solution (1% Coomassie Brilliant Blue R-250, 40% methanol, 10% acetic acid) and destained in the same solvent (40% methanol, 10% acetic acid) for 6 h. The Invitrogen iBright™ FL1500 Imaging System (Thermo Fisher Scientific) was used to visualize the presence of the MMPs. Intensity of the bands were quantified using Fiji [32].

#### Statistics

Statistical analyses were performed using GraphPad Prism version 10.0.0 for Windows (GraphPad Software, Boston, Massachusetts, USA). Gene expression and compaction data are represented as mean and standard deviation. For the tissue compaction results, comparisons were made between different groups at 72 h after a normality check. For the gene expression results, comparisons were made between different groups for the same gene after a normality check. If the distribution was normal, a One-Way ANOVA test with Tukey post hoc test was used. If the distribution was not normal, a Kruskal–Wallis test with Dunn post hoc test was used. For the multiplex ELISA results, comparisons were made between the paired data after 24 and 48 h using a Wilcoxon test. Differences were considered statistically significant when* p* < 0.05.

## Results

### Polarised macrophages show different secretion profiles over time

**Fig. 4:**
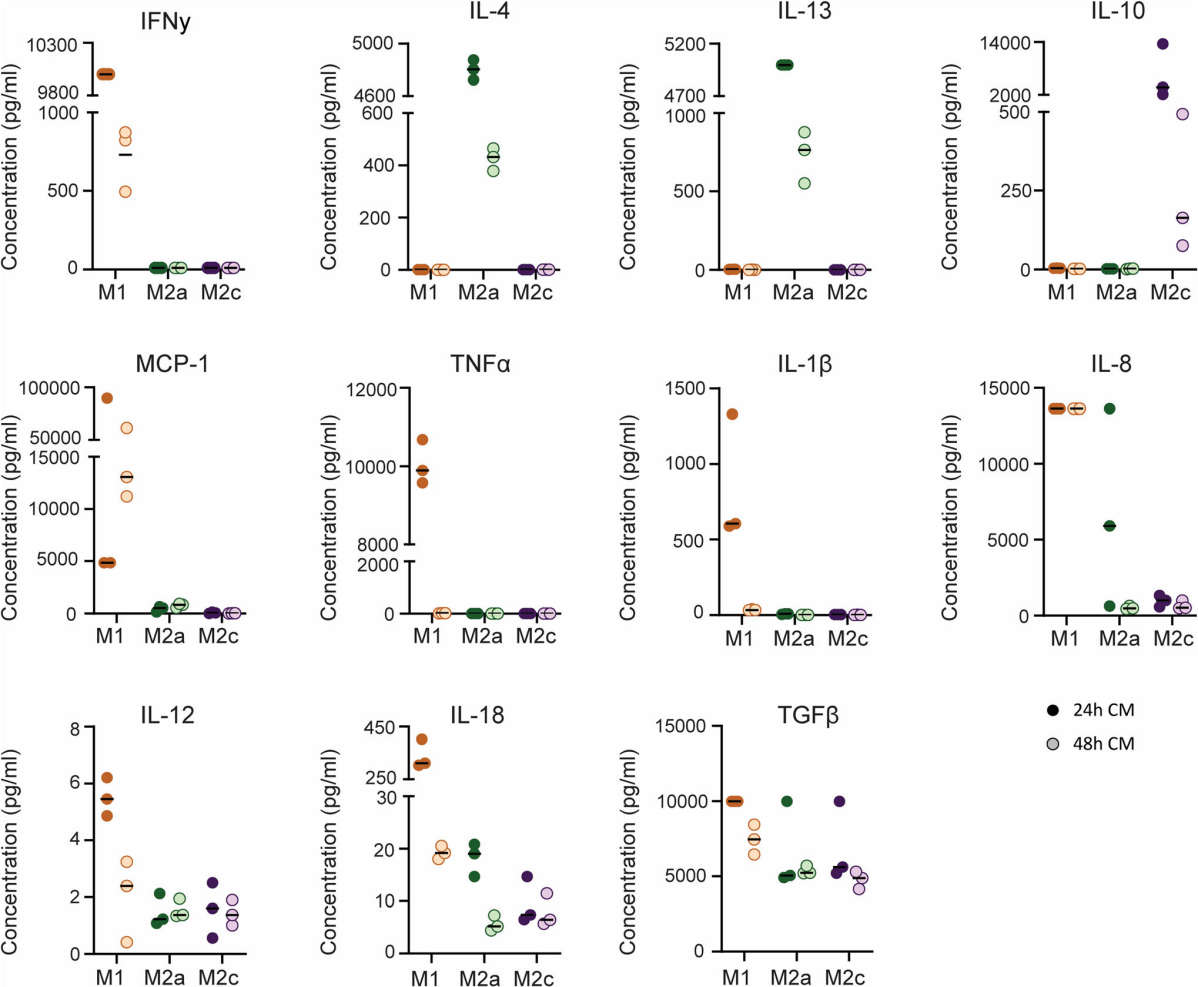
Quantification of signalling factors in the medium after 24 h and 48 h. The 24 h samples contain the added polarising factors (M1: IFN-γ, M2a: IL-4 and IL-13 and M2c: IL-10) in addition to cell-secreted factors, while the 48 h samples only contain factors secreted by the cells between 24 and 48 h. N = 3 for each condition

Conditioned medium from macrophages was collected after 24 h (24 h CM), when full polarization was achieved. Fresh culture medium without PFs was added, to exclude the possible influence of the PFs, and the second batch of CM was collected another 24 h later, resulting in 48 h CM (Fig. 1). Polarising factors could still be detected after 24 h of incubation with macrophages (24 h CM; Fig. 4). A substantial decrease in PF concentrations in 48 h CM compared to 24 h CM was observed (Fig. 4). M1 polarisation resulted in early secretion of pro-inflammatory cytokines TNFα, IL-1β, IL-18, and, to a lesser extent, IL-12 within the first 24 h of culture (24 h CM), but not between 24 and 48 h (48 h CM), while late secretion of IL-8 and MCP-1 by M1 macrophages was sustained at 48 h. These differences were not as striking in the M2a and M2c groups, with generally low secretion levels of the pro-inflammatory cytokines. Notably, TGF-β was secreted by all macrophage types at both the early and late time points (Fig. 4).

### 24 h CM and 48 h CM affect tissue compaction and gene expression levels differently

**Fig. 5:**
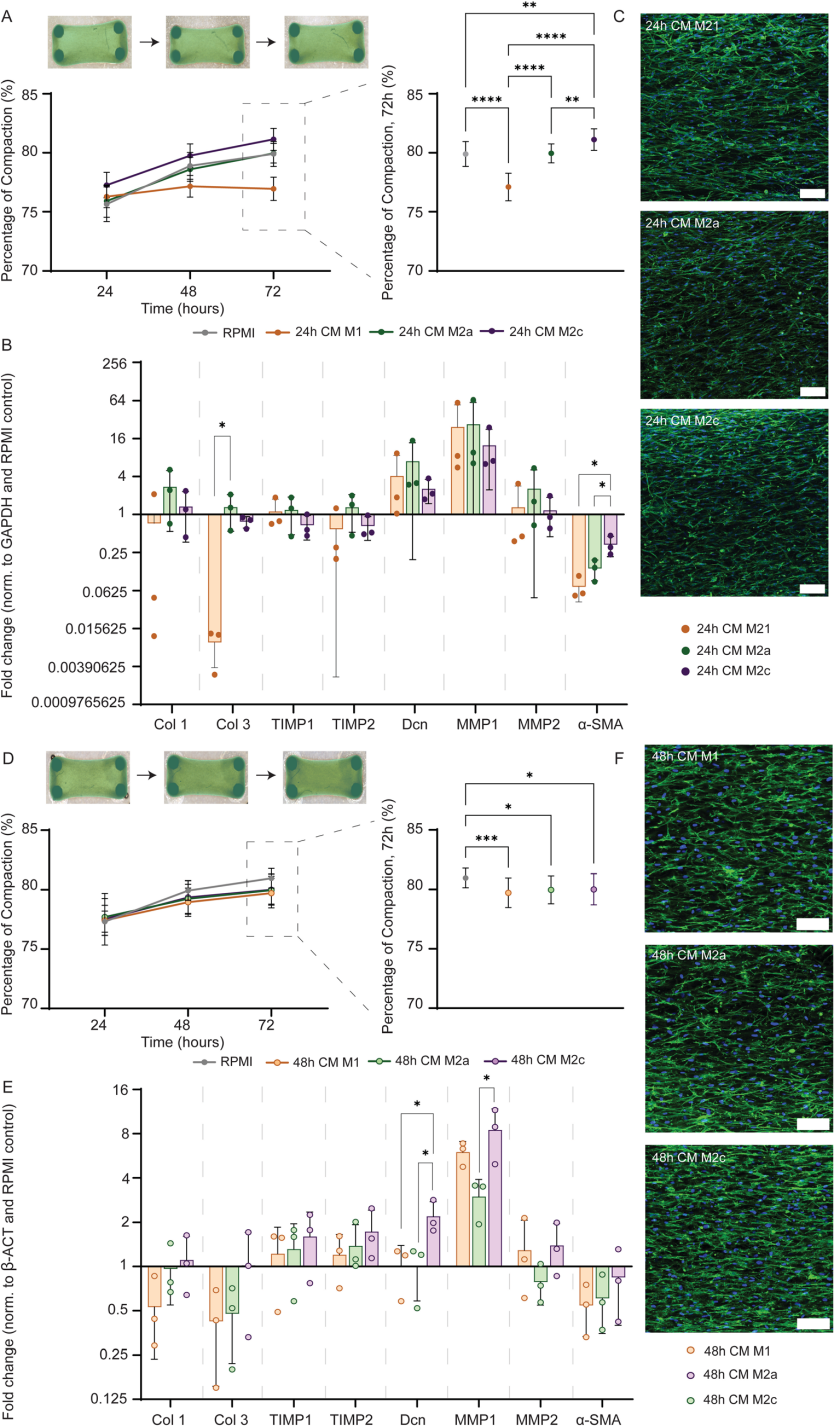
Conditioned medium obtained from differently polarized macrophages after 48 h (in absence of polarizing factors) had comparable effects on tissue compaction, gene expression and morphology of cells. **A** Percentage of tissue compaction at 24, 48 and 72 h of tissues stimulated with M1, M2a, or M2c 24 h CM, calculated using masking in green. Statistical analysis was performed on 72 h data. N = 17–20 for each condition. **B** Gene expression profiles of fibroblasts in M1, M2a and M2c 24 h CM stimulated tissues. Fold changes in relative expression of production, degradation and contraction related genes when compared to the RPMI control. N = 3 for each condition. **C** Representative fluorescent images of the phalloidin and DAPI staining visualizing the cell morphology at 72 h of 24 h CM stimulated tissues (whole mount staining, maximum intensity projection, green = phalloidin; blue = DAPI). **D** Percentage of tissue compaction at 24, 48 and 72 h when stimulated with M1, M2a, M2c 48 h CM, calculated using masking in green. Statistical analysis was performed on 72 h data. N = 26 for each condition. **E** Gene expression profiles of fibroblasts in M1, M2a and M2c 48 h CM stimulated tissues. Fold changes in relative expression of production, degradation and contraction related genes when compared to the RPMI control. N = 3 for each condition. **F** Representative fluorescent images of the phalloidin and DAPI staining visualizing the cell morphology at 72 h of 48 h CM stimulated tissues (whole mount staining, maximum intensity projection, green = phalloidin; blue = DAPI). Scale bars, 100 µm. *p < 0.05, **p < 0.01, ***p < 0.001, ****p < 0.0001. CM: conditioned medium

Culturing the tissues in 24 h CM resulted in significant differences in tissue compaction between all conditions, with M1 24 h CM causing the lowest tissue compaction and M2c 24 h CM causing the highest tissue compaction (Fig. 5A). However, no significant differences were found in gene expression levels between groups, except for collagen 3 and α-SMA (Fig. 5B). No clear differences in morphology or orientation of the cells were observed (Fig. 5C).

No significant differences in compaction between the different 48 h CM groups were observed, suggesting that the sustained late secreted factors (i.e. MCP-1 and IL-8) are not the main responsible factors for tissue compaction (Fig. 5D). However, all the CMs resulted in a significant reduction in tissue compaction, compared to the control. Additionally, no significant differences were found in gene expression levels between groups, except for Dcn and MMP-1 (Fig. 5C). Finally, no clear differences in morphology or orientation of the cells were observed (Fig. 5D).

### Polarising factors directly affect tissue compaction

**Fig. 6:**
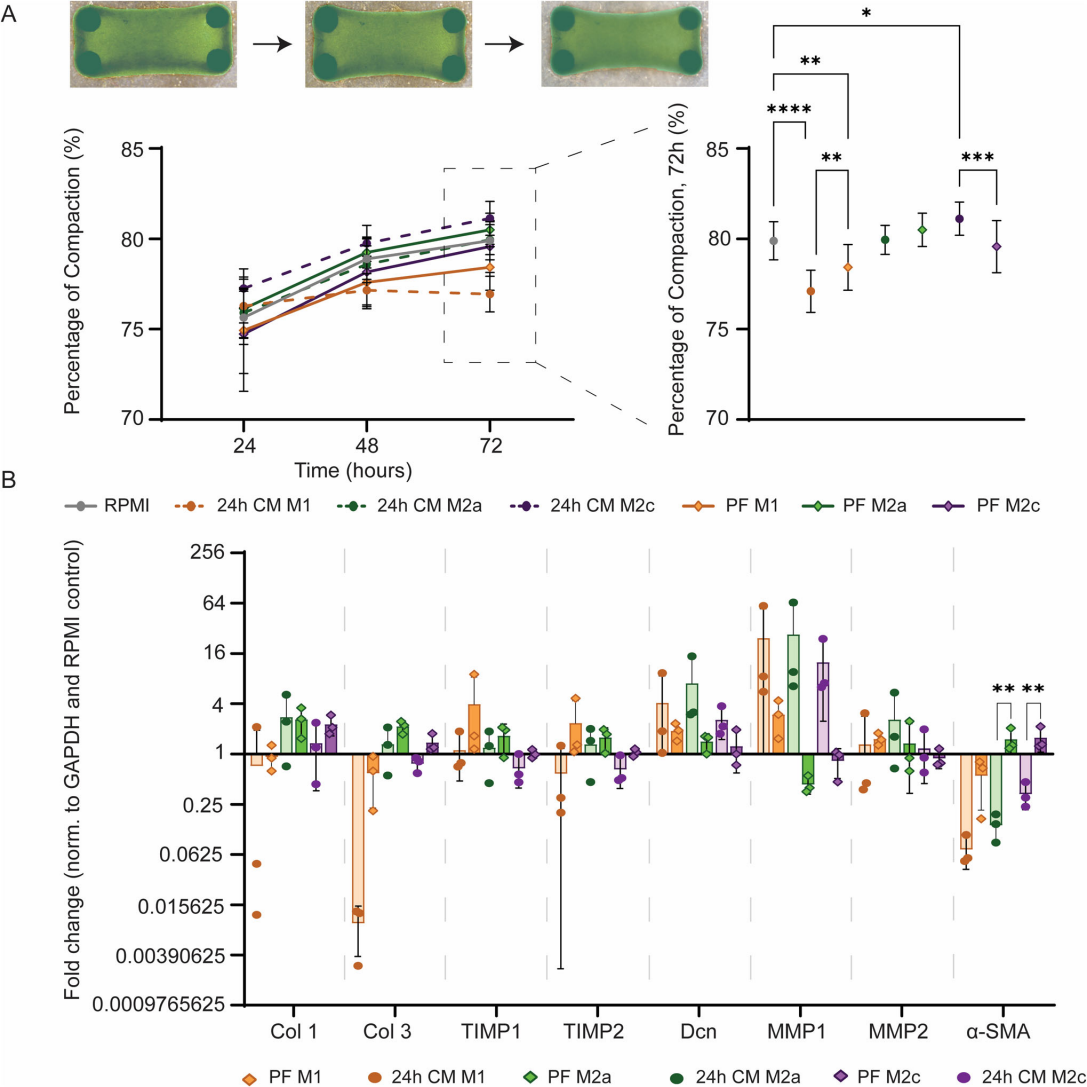
Polarising factors influenced tissue compaction and gene expression. **A** Percentage of tissue compaction at 24, 48 and 72 h of tissues stimulated with M1, M2a, or M2c 24 h PF compared to 24 h CM, calculated using masking in green. Statistical analysis was performed on 72 h data. N = 17–20 for each condition. **B** Gene expression profiles of fibroblasts in M1, M2a and M2c 24 h PF stimulated tissues compared to 24 h CM stimulated tissues. Fold changes in relative expression of production, degradation and contraction related genes when compared to the RPMI control. N = 3 for each condition. *p < 0.05, **p < 0.01, ***p < 0.001, ****p < 0.0001. PF: Polarising Factors; CM: conditioned medium

Given the presence of high concentrations of PFs in the 24 h CM, it was assessed whether the differences in tissue compaction and gene expression were directly caused by the presence of PFs. M1 PFs (LPS, IFN-γ) have the strongest effect on fibroblast behaviour, as shown by the significant difference in tissue compaction compared to RPMI and the upregulation of TIMP-1, TIMP-2, MMP-1 and the downregulation of α-SMA. M2a and M2c PFs have a lower influence as shown by the lack of significant differences in tissue compaction. However, α-SMA gene expression is slightly upregulated compared to M1 PF. While the PFs clearly influence tissue compaction and gene expression, they do not result in the same outcomes as the corresponding 24 h CM groups. The compaction of PF M1 and PF M2c stimulated tissues was significantly different in comparison to 24 h CM M1 and 24 h CM M2c stimulated tissues, respectively (Fig. 6A). Similarly, the α-SMA gene expression of the M2a and M2c PF stimulated tissues also significantly differed from the 24 h CM M2a and 24 h CM M2c stimulated tissues (Fig. 6B). Overall, this indicates that the remaining differences are caused by other factors present in the medium.

### Cytokine-enriched media serum levels affect tissue compaction

In order to further explore the effect of early macrophage secreted factors on fibroblast-induced tissue compaction, isolated from the observed confounding effects of PFs, defined cytokine-enriched media (CE) were produced based on the quantified cytokine concentrations at 24 h for each of the different macrophage subsets (Table 4). Media were produced with high (10%) or low (2.5%) levels of serum to probe the potential effect of unknown serum factors, which may mask the effects of macrophage-secreted factors.

**Table 4:** Concentration of secreted factors detected in conditioned medium harvested at 24 h by multiplex-ELISA, used to create simulated conditioned medium

ng/mL	TNF-α	IL-1β	IL-12	IL-8	IL-18	MCP-1	TGF-β
M1	10.052	0.842	0.006	13.637	0.339	89.730	9.999
M2a	0.001	0.008	0.001	6.724	0.018	0.451	4.985
M2c	0.001	0.005	0.002	0.974	0.010	0.068	6.941

**Fig. 7:**
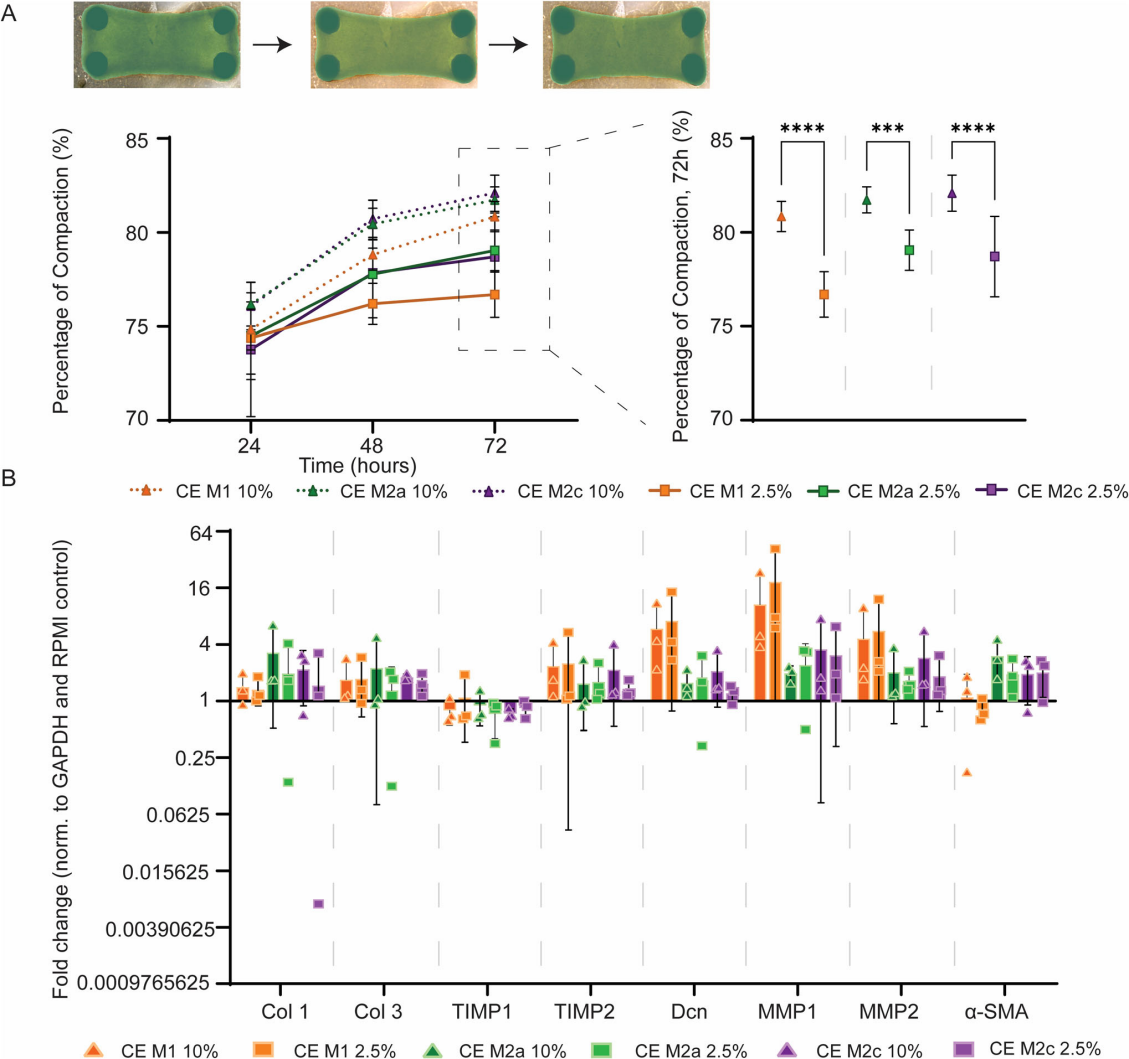
Secreted factors in the cytokine-enriched media affected tissue compaction and gene expression. Medium serum levels influenced tissue compaction, but shows equal trends in gene expression profiles.** A** Percentage of tissue compaction at 24, 48 and 72 h, stimulated with M1, M2a, M2c CE medium containing 2.5% or 10% FBS, calculated using masking in green. Statistical analysis was performed on 72 h data. N = 16–20 for each condition. **B** Gene expression profiles of fibroblasts in M1, M2a and M2c CE medium containing 2.5% or 10% FBS. Fold changes in relative expression of production, degradation and contraction related genes, compared to the RPMI control. N = 3 for each condition. *p < 0.05, **p < 0.01, ***p < 0.001, ****p < 0.0001. CE: cytokine-enriched medium

For all macrophage types, the use of low serum led to significantly less compaction when compared to high serum levels (Fig. 7A). This was not reflected in any significant differences at gene level between serum concentrations, for any macrophage subset (Fig. 7B). Due to the significant differences in tissue compaction between high and low serum levels, indicating a potential effect of serum factors on our read-outs, low serum level data were used for further interpretation.

### MMP production and cellular contraction forces by resident fibroblasts may underlie lower tissue compaction caused by M1 cytokine-enriched medium compared to M2a/M2c cytokine-enriched media

**Fig. 8:**
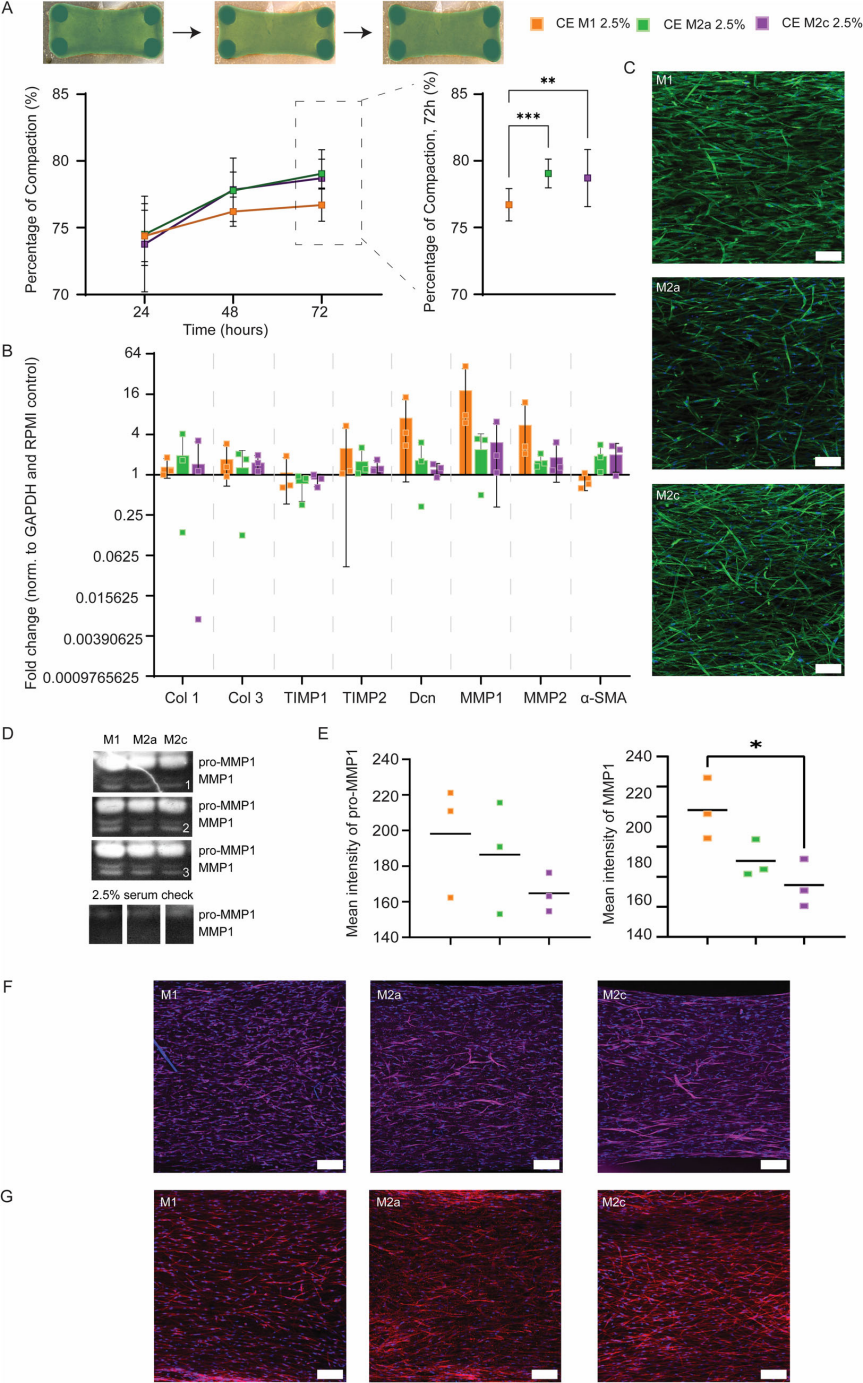
Secreted factors in the 2.5% Cytokine-enriched medium affected tissue compaction and gene expression. MMPs and cellular contraction are present in collagenous tissues stimulated with 2.5% CE medium. **A** Percentage of tissue compaction at 24, 48 and 72 h, stimulated with M1, M2a, M2c CE medium containing 2.5% serum, calculated using masking in green. Statistical analysis was performed on 72 h data. N = 16–20 for each condition. **B** Gene expression profiles of fibroblasts in M1, M2a and M2c CE medium containing 2.5% serum; fold changes in relative expression of production, degradation and contraction related genes, compared to the RPMI control. N = 3 for each condition. **C** Representative fluorescent images of the phalloidin and DAPI staining visualizing the cell contractility at 72 h (whole mount staining, maximum intensity projection, green = phalloidin; blue = DAPI) **D** Detected collagenases, pro-MMP1 and MMP1, in culture medium obtained from M1, M2a and M2c CE medium (2.5% FBS) stimulated tissues at 72 h. **E** Quantified intensities of detected pro-MMP1 and MMP1. Statistical analysis was performed on 72 h data. N = 3 for each condition. **F** Representative fluorescent images of the α-SMA and DAPI staining visualizing the cell contractility at 72 h (whole mount staining, maximum intensity projection, magenta = α-SMA; blue = DAPI). Scale bars, 100 µm. **G** Representative fluorescent images of the phosphorylated myosin and DAPI staining visualizing the cell contractility at 72 h (whole mount staining, maximum intensity projection, red = phosphorylated myosin; blue = DAPI). Scale bars, 100 µm. *p < 0.05, **p < 0.01, ***p < 0.001, ****p < 0.0001. CE: cytokine-enriched

Tissue compaction by all cytokine-enriched media began at similar levels at 24 h. By 72 h, M1 cytokine-enriched medium showed significantly reduced compaction compared to M2a/M2c cytokine enriched media (Fig. 8A). Despite no significant differences, a trend of higher expression of TIMP2, Dcn, MMP1 and MMP2 and a lower expression of α-SMA were observed in tissues stimulated with M1 cytokine-enriched medium, while the expression of Collagen 1, Collagen 3 and TIMP1 was similar in all tissues (Fig. 8B). Qualitatively, no differences were observed in the morphology of the cells and their orientation (Fig. 8C). To identify the underlying mechanisms causing the observed differences in tissue compaction, we analysed cell contractility and tissue degrading enzymes secreted by the fibroblasts treated with the specific macrophage-associated cytokine-enriched media. Collagen zymography was performed to detect the presence of MMP1, compared to expansion medium with only 2.5% serum as a control (Fig. 8D). Tissues were stained for α-SMA and phosphorylated myosin to visualize smooth muscle actin and myosin, as a measure for cellular contractility (Fig. 8F, G). Two bands at MMP1 (~ 46 kDa) and pro-MMP1 (~ 59 kDa) were detected at a significantly higher intensity in the M1, M2a, and M2c groups compared to the 2.5% serum control, indicating the presence of secreted collagenases (Fig. 8E). The intensity of the MMP1 secreted by fibroblasts in M1 cytokine-enriched medium was significantly higher than fibroblasts in M2c cytokine-enriched medium, indicating that the cells potentially shifted towards a catabolic phenotype. α-SMA fibres and active stress fibres, seen by the phosphorylated myosin, were present in all three experimental groups at qualitatively corresponding intensities. Qualitatively, M2c samples show more profound α-SMA fibres than M1 and M2a samples (Fig. 8G).

## Discussion

This study aimed to elucidate how paracrine signalling, from different macrophage phenotypes, affects tissue remodelling ability of fibroblasts, particularly through the compaction of their surrounding collagenous matrix. To achieve our aim, the effect of conditioned medium collected from differently polarised macrophages -either directly, or via administration of the most prominent secreted factors by macrophages in cytokine-enriched media- was investigated. Our results show that factors secreted by M1 macrophages, compared to M2a/M2c macrophages, reduce tissue compaction by resident fibroblasts.

Addressing our aim involved optimising the methodology for producing macrophage conditioned medium (CM) that solely captures the effect of the cell-secreted factors, i.e., true paracrine signalling, in absence of confounding factors. The conditions under which conditioned medium is made are often overlooked in many studies, as stated by Hult et al. [34]. Whereas several studies have PFs present in their CM [35, 36], others explicitly ensure that the CM used in their experimental groups are free of PFs [22, 37–41]. Interestingly, it is known that, for example, the M1 polarising factor LPS can have a direct effect on the proliferative and collagen synthesis ability of fibroblasts [42]. Both IFN-γ and IL-10 are considered to be master regulators of many inflammatory and regulatory pathways, including the inhibition of TGF-β production and collagen production [43–46]. The M2a PFs IL-4 and IL-13, on the other hand, have been implicated with myofibroblast activation and collagen synthesis [44, 47, 48]. Hence, the prolonged presence of these factors can potentially skew results. The study by Hult et al., [34] revealed that the profibrotic effects associated with M2 macrophage CM *in vitro* are attributable to effects of the PFs, rather than factors secreted by polarised macrophages. This is in line with our observations that revealed that the PFs used *in vitro* to induce differentiation of macrophages into M1 or M2 phenotypes significantly influenced tissue compaction and gene expression compared to the RPMI control group. Therefore, caution should be paid to the method by which conditioned medium is prepared to study paracrine signalling, since it can influence the read-outs. Removal of polarizing factors before obtaining the conditioned medium, analogous to other studies [22, 40], however, severely reduced the concentration of early secreted factors by macrophages, thereby eliminating effects on tissue compaction. For example, TNF-α is known to be predominantly secreted within the first 24 h after LPS stimulation [49], and, hence, is no longer present in the 48 h sample after medium change in our current setup, which is consistent with our current data. Interestingly, the aforementioned studies did report effects of the different conditioned media on other read-outs such as cell migration and phenotype shift [22, 40].

Next, the change from high to low serum levels, as part of the conditioned medium, reduced tissue compaction, in analogy to previous research [50]. High serum levels, commonly used in cell culture studies [51], contain high levels of growth factors and matrix degrading proteins, e.g., TGF-β and MMPs, that directly affect cell-induced tissue compaction. A reduction in serum concentration, an approach also adopted by Perdiguero et al. [50], significantly reduced tissue compaction for all groups.

Ultimately, defined cocktails of prominent secreted factors at the correct concentrations, associated with the various macrophage subsets, were used in the current study to assess paracrine signalling by macrophages on tissue remodelling features. The selected signalling factors include TGF-β, which is known to activate α-SMA expression in fibroblasts and has long been the hallmark of fibrosis [52, 53]. TGF-β is often associated with an M2 macrophage response, however, our results show that TGF-β is not distinctive to a particular macrophage subset and that all macrophage subsets secrete abundant levels of TGF-β, which is consistent with previous reports [54]. Other included factors are the pro-inflammatory factors TNF-α and IL-1β, which have been pinpointed as pro-fibrotic factors via the induction of TGF-β [29, 55]. Moreover, IL-1β in combination with IL-18, are the products of the NLRP3 inflammasome activation, which has been directly implicated with fibrosis as well [30]. MCP-1 and IL-8 were detected in both M1- and M2a conditioned medium, albeit at lower concentrations in the M2a medium, and hence included in the respective cytokine-enriched media as well. MCP-1 and IL-8 are primarily strong chemoattractants for monocytes and neutrophils, respectively [56, 57]. However, both factors have been described to have direct effects on fibroblasts as well, including the promotion of fibroblast chemotaxis [58]. Additionally, MCP-1 has been shown to promote collagen production by fibroblasts, via the upregulation of endogenous TGF-β [59]. The final cytokine included was IL-12, which, in contrast to the other factors, has been linked to the inhibition of fibrosis, via the promotion of IFN-γ [60].

By carefully curating and selecting the secreted factors, we could show that the most important factors were captured to mimic macrophage-derived conditioned medium, as only minor differences existed between conditioned medium and a selection of secreted factors per group (Supplementary Fig. 2). Furthermore, it demonstrates that our approach of composing conditioned medium can be effective. However, we acknowledge certain limitations in our selection and CE curation methodology. For example, by using macrophage-conditioned/-enriched media, we focused on the signalling from specific macrophage subsets to fibroblasts only, thereby neglecting reciprocal signalling from fibroblasts to macrophages. Since such signalling pathways may be synergistic, we may have over- or underestimated the influence of certain cytokines [55, 61]. Moreover, the panel of cytokines measured with ELISA was based on the expected potential links between macrophage polarization and fibroblast regulation, but this panel is not exhaustive. Most notably, IL-6 was not included in our ELISA analysis because THP-1-derived macrophages have previously been reported to lack secretion of IL-6, in contrast to primary macrophages [62]. IL-6 is secreted by both macrophages and fibroblasts via a positive feedback loop and the IL-6-dependent macrophage-fibroblast signalling pathway has been implicated with fibrosis [63–65]. Specifically, IL-6 is secreted by macrophage-stimulated fibroblasts and it has been shown that IL-6 stimulated myofibroblast activation and collagen synthesis in various fibrotic conditions [63–65]. Hence, its absence in the present study may have influenced the biological activity of our CE medium. We utilised the THP-1 cell line, instead of for example primary human macrophages, due to its robustness and absence of donor variability [66]. While this choice offers consistency, it also introduces key differences when compared to primary cells, particularly in gene expression and cytokine secretion profiles [27, 54, 62, 66].

Furthermore, apart from other proteins that we may have overlooked, the conditioned medium did not include other relevant factors that macrophages are known to secrete, such as extracellular vesicles (EVs) and lipids [67, 68]. For example, Qin et al. observed that EVs isolated from silica-exposed macrophages are profibrogenic [69]. Hua et al. report that large EVs from macrophages transfer mitochondria to promote adipocyte–myofibroblast transition in epidural fibrosis [68]. To be complete, these should be added to the cytokine-enriched medium. An all-encompassing technique to analyse the complete secretome, such as proteomics and mass spectrometry, may help to identify relevant such factors. Once all relevant factors are identified, tailored cytokine-enriched media can potentially be developed to investigate paracrine phenomena, allowing for more consistent comparisons across different studies using the same media.

Continuing with our developed approach, we hypothesized that the pro- and anti-inflammatory factors would influence the tissue remodelling ability of fibroblasts. Increased compaction of the collagen gel would indicate a higher potential for fibroblasts to rearrange their matrix structurally. Compaction of our tissues is expected to be caused by two main mechanisms: fibroblast-induced degradation and contraction of the collagen environment. The ECM is degraded during the initial phase of remodelling, while in the later phase, new ECM is produced and reorganised [70]. It is known that macrophages secrete collagenases such as MMPs that degrade the ECM, and in addition, they can secrete factors that stimulate fibroblasts to release collagenases [71]. Cellular contraction, initiated by the cytoskeletal network, exerts forces on the ECM through which they reorganise ECM [72]. This fibroblast-mediated ECM tension is known to contribute to repair, since increased cellular contractile forces aid in collagen fibre rearrangement [73]. Fibroblasts are expected to be less contractile in the early remodelling phase and more contractile in the later phase when new collagen is laid down and aligned [19, 22].

Correspondingly, our experiments revealed that M1 cytokine-enriched medium resulted in significantly less tissue compaction compared to M2a/M2c cytokine-enriched media. Matrix degradation-related genes MMP1 and MMP2, a collagenase and gelatinase, and Dcn, which contributes to establishing tissue anisotropy, were expressed highly in the M1 CM conditions. In accordance with the gene expression, collagen zymography revealed the highest activity of MMP1 for M1 CM conditions. Although no significant differences were found in our selected contractility readouts, i.e., α-SMA gene expression and the stains of both α-SMA and phosphorylated myosin, the ability of the fibroblast to bind and retract collagen might have been reduced [74, 75]. Specifically, the concentrations of TNF-α, IL-8, and MCP-1 in M1 cytokine-enriched medium were higher than in the M2a/M2c cytokine-enriched medium (Table 4). For example, IL-8 was found to reduce the expression of focal adhesions in fibroblasts [58], and TNF-α decreased F-actin polymerisation and led to more star-shaped cells with reduced cytoplasmic processes [76]. Hence, the lower tissue compaction could be a combined effect of the cell’s increased degradative activity leading to a looser ECM network and their reduced ability to reorganize this surrounding matrix. This could indicate that the fibroblasts are degrading the surrounding collagen fibres and subsequently making space for other cells and new tissue to be formed, which is in line with M1 macrophages contributing to the first phase of tissue healing [18, 71]. This could imply that the prolonged presence of M1 macrophages could hinder the functional remodelling process. Due to the use of human dermal fibroblasts in this study, our model and results can be applied to various collagenous tissues. For example, it has been shown to be a representative fibroblast cell type for other fibroblasts such as tenocytes [77].

Using this microtissue platform, we successfully generated microtissues in a robust and controlled manner, enabling the investigation of fibroblast behaviour during tissue reorganization. Despite its advantages, the platform presents certain limitations, particularly regarding its representation of *in vivo* tissue remodelling. Specifically, cell-mediated processes such as collagen synthesis, degradation, and dynamic reorganization are not adequately captured within the short experimental timeframe. Instead, the observed phenomena primarily reflect cellular responses to mechanical constraints imposed by the posts, resulting in cell alignment and subsequent reorientation of collagen fibrils. This onset of reorganization can be different from the cues that initiate tissue remodelling* in vivo* such as dynamic biomechanical cues (e.g. cyclic stretch) and long-term remodelling. The behaviour we observed does align with existing literature suggesting that cell-generated traction forces can induce substantial tissue pre-stretch, thereby contributing to the formation of anisotropic tissue structures [78]. Therefore, the model is suited to study the cell’s ability to remodel their surrounding ECM.

In conclusion, our data indicate that factors secreted by M1 macrophages, compared to M2a/M2c macrophages, result in reduced tissue compaction in an *in vitro* model of tissue remodelling, indicating a reduced capacity for fibroblasts to remodel the ECM. In addition, our study highlights that paracrine signalling studies should be designed and developed while carefully considering, and preferably analysing, the factors that are present in the conditioned medium, as polarising factors and serum can directly influence the cells and hence can warp the results observed.

## Supplementary Information

Below is the link to the electronic supplementary material.

Supplementary file1 (DOCX 586 kb)

## Data Availability

The datasets generated during and/or analysed during the current study are available from the corresponding author on reasonable request.
