# Supplementary material for: Macrophage Paracrine Signalling Differentially Affects Fibroblast-Induced Collagenous Tissue Remodelling
Source: Tissue Eng Regen Med. 2025 Nov 28;23(1):125–42. doi: 10.1007/s13770-025-00766-1 (PMC12775218; doi:10.1007/s13770-025-00766-1)
Supplement: Supplementary file 1 — Supplementary file1 (DOCX 586 kb) [file 13770_2025_766_MOESM1_ESM.docx]

# Macrophage paracrine signalling differentially affects fibroblast-induced collagenous tissue remodelling

# **Supplementary information**

**
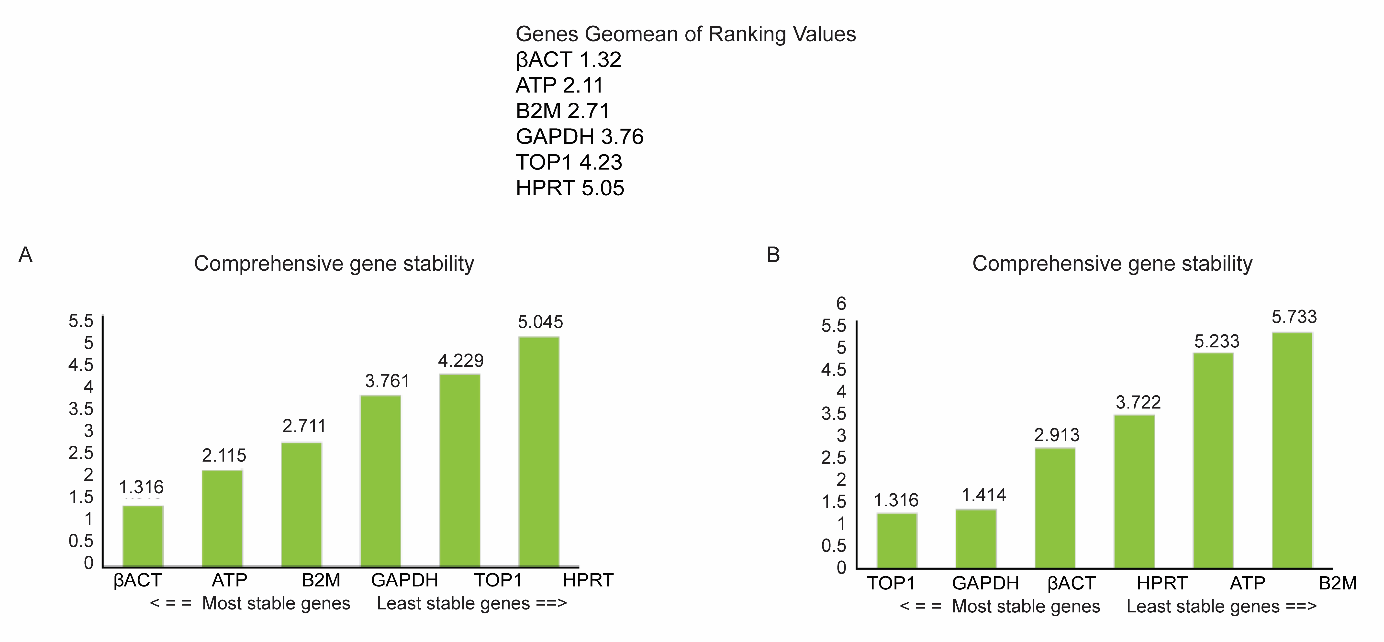
**

**Supplementary Fig. 1**. **Ranking of the reference genes tested for stability in RT-qPCR analysis.** **A** Analysis was performed on 48h CM and RPMI control groups, **B** Analysis was performed on PF, CE, CE+PF, CE 2.5%, 24h CM and RPMI control groups. N=3


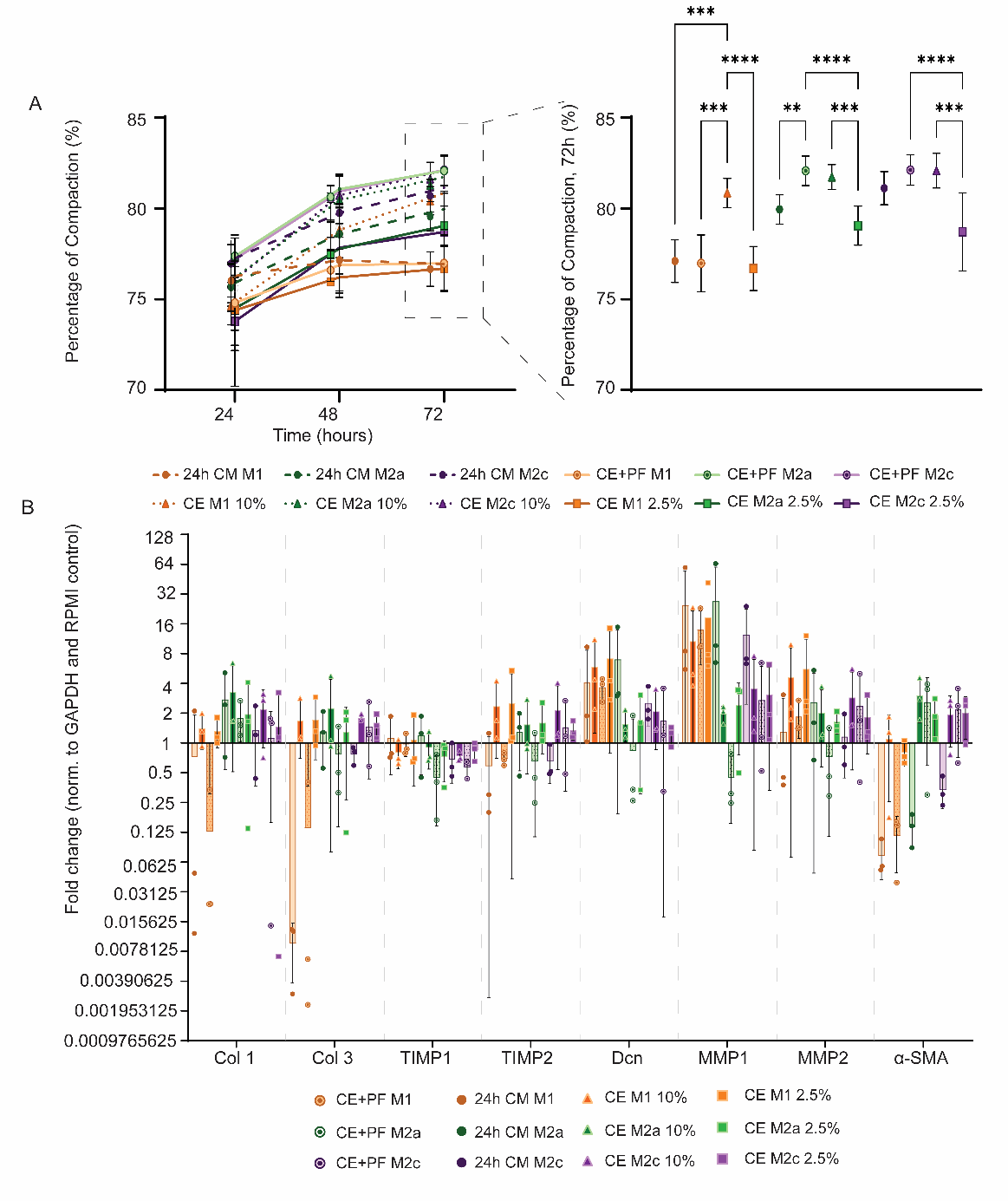


**Supplementary Fig. 2. A comparison between conditioned (24h CM) media that was produced directly from macrophages and also contained PFs shows minor difference between cytokine-enriched + polarising factor media. A** Percentage of tissue compaction at 24, 48 and 72 hours, tissues stimulated with M1, M2a, M2c 24h CM, PF, 2.5% CE, 10% CE and CE+PF medium, calculated using masking in green. Statistical analysis was performed on 72h data. N=16-21 for each condition. **B** Gene expression profiles of fibroblasts in M1, M2a, M2c 24h CM, PF, 2.5% CE, 10% CE and CE+PF medium. Fold changes in relative expression of production, degradation and contraction related genes, compared to the RPMI control. N=3 for each condition; CE: Cytokine-enriched, PF: Polarising Factors

***Supplementary Table 1.*** *Protein secretion profiles of polarized macrophages (M1, M2a, M2c) after 24 and 48 hours, measured using Multiplex ELISA. N=3*

|  | **IFNg (53)** | **IL4 (34)** | **IL13 (39)** | **IL10 (37)** | **IL12 (38)** | **TGFb (58)** | **MCP1 (54)** | **IFNg (53)** | **TNFa (60)** | **IL1b (12)** | **IL8 (212)** | **IL18 (29)** |
| --- | --- | --- | --- | --- | --- | --- | --- | --- | --- | --- | --- | --- |
| **Description** | **pg/ml** | **pg/ml** | **pg/ml** | **pg/ml** | **pg/ml** | **pg/ml** | **pg/ml** | **pg/ml** | **pg/ml** | **pg/ml** | **pg/ml** | **pg/ml** |
| M1 24h (1) | OOR > | 1,13 | 3,35 | 4,69 | 5,46 | OOR > | OOR > | OOR > | *9576,51 | 1330,15 | OOR > | 401,84 |
| M1 24h (2) | OOR > | 1,14 | 4,71 | 3,81 | 6,21 | OOR > | *89730,46 | OOR > | *9895,19 | 606,20 | OOR > | 311,83 |
| M1 24h (3) | OOR > | 1,30 | 3,70 | 3,64 | 4,86 | OOR > | OOR > | OOR > | *10683,06 | 588,85 | OOR > | 303,82 |
| M1 48h (1) | 494,67 | *0,63 | OOR < | OOR < | *3,24 | 6461,19 | *11199,97 | 494,67 | 11,36 | 39,78 | OOR > | 18,02 |
| M1 48h (2) | 823,19 | *0,51 | 1,46 | OOR < | *0,42 | 7457,11 | *13054,81 | 823,19 | 19,28 | 33,31 | OOR > | 19,16 |
| M1 48h (3) | 873,48 | *0,25 | OOR < | OOR < | *2,39 | 8449,18 | *61459,10 | 873,48 | 19,42 | 34,03 | OOR > | 20,49 |
|  |  |  |  |  |  |  |  |  |  |  |  |  |
| M2A 24h (1) | OOR < | *4874,86 | OOR > | OOR < | *2,12 | OOR > | 160,20 | OOR < | OOR < | 6,03 | 626,62 | 14,67 |
| M2A 24h (2) | OOR < | *4803,68 | OOR > | OOR < | *1,08 | 5054,93 | 539,70 | OOR < | OOR < | 8,99 | 5908,84 | 19,03 |
| M2A 24h (3) | OOR < | *4722,48 | OOR > | OOR < | *1,23 | 4915,70 | 653,16 | OOR < | OOR < | 9,15 | OOR > | 20,81 |
| M2A 48h (1) | OOR < | 378,34 | 551,58 | *0,86 | *1,94 | 5207,09 | 553,25 | OOR < | OOR < | 2,28 | 638,49 | 7,27 |
| M2A 48h (2) | OOR < | 465,03 | 764,46 | 2,77 | *1,34 | 5225,48 | 838,56 | OOR < | OOR < | 1,28 | 447,40 | 4,43 |
| M2A 48h (3) | OOR < | 431,95 | 877,73 | 2,52 | *1,38 | 5703,41 | 920,31 | OOR < | OOR < | 1,90 | 482,96 | 5,17 |
|  |  |  |  |  |  |  |  |  |  |  |  |  |
| M2C 24h (1) | OOR < | 0,82 | OOR < | 1999,14 | *1,60 | OOR > | 17,80 | OOR < | OOR < | 4,61 | 585,92 | 14,67 |
| M2C 24h (2) | OOR < | 1,03 | OOR < | 3627,82 | *2,50 | 5207,40 | 59,96 | OOR < | OOR < | 4,15 | 1011,47 | 6,47 |
| M2C 24h (3) | OOR < | 1,08 | OOR < | *13592,78 | *0,56 | 5617,59 | 126,92 | OOR < | OOR < | 4,97 | 1326,01 | 7,36 |
| M2C 48h (1) | OOR < | 0,90 | OOR < | 75,17 | *1,01 | 4152,35 | 17,88 | OOR < | OOR < | 3,10 | 999,85 | 11,46 |
| M2C 48h (2) | OOR < | 0,84 | OOR < | 163,27 | *1,38 | 4887,06 | 21,50 | OOR < | OOR < | 1,65 | 495,51 | 5,68 |
| M2C 48h (3) | OOR < | 0,94 | OOR < | 492,81 | *1,90 | 5300,47 | 29,11 | OOR < | OOR < | 2,15 | 524,20 | 6,43 |

***Supplementary Table 2****. Upper and lower cut-off values of multiplex ELISA measurements*

| *Protein* | *Symbol* | *ELISA cut-off values (pg/mL)* |
| --- | --- | --- |
| Monocyte chemoattractant protein 1 | MCP-1 | 0,973 - 4844,687 |
| Interferon gamma | IFN-γ | 10,662  - 9999,949 |
| Tumor necrosis factor alpha | TNF-α | 1,392  - 4978,677 |
| Interleukin 1 beta | IL-1β | 1,190  - 4997,159 |
| Interleukin 4 | IL-4 | 0,659   - 2421,576 |
| Interleukin 8 | IL-8 | 4,555   - 13636,916 |
| Interleukin 10 | IL-10 | 2,043  - 10405,870 |
| Interleukin 12 p70 | IL-12 p70 | 4,668  - 20067, 926 |
| Interleukin 13 | IL-13 | 1,176  - 4996,382 |
| Interleukin 18 | IL-18 | 1,193   - 5006,347 |
| Transforming growth factor beta 1 | TGF-β1 | 1,526   - 9998,818 |
